# Gaussian Process Panel Modeling—Machine Learning Inspired Analysis of Longitudinal Panel Data

**DOI:** 10.3389/fpsyg.2020.00351

**Published:** 2020-03-19

**Authors:** Julian D. Karch, Andreas M. Brandmaier, Manuel C. Voelkle

**Affiliations:** ^1^Methodology and Statistics, Institute of Psychology, Leiden University, Leiden, Netherlands; ^2^Formal Methods in Lifespan Psychology, Center for Lifespan Psychology, Max Planck Institute for Human Development, Berlin, Germany; ^3^Max Planck UCL Centre for Computational Psychiatry and Ageing Research, Berlin, Germany; ^4^Psychological Research Methods, Department of Psychology, Humboldt University of Berlin, Berlin, Germany

**Keywords:** longitudinal analysis, machine learning, statistical learning, Bayesian, continuous-time, prediction

## Abstract

In this article, we extend the Bayesian nonparametric regression method *Gaussian Process Regression* to the analysis of longitudinal panel data. We call this new approach *Gaussian Process Panel Modeling (GPPM)*. GPPM provides great flexibility because of the large number of models it can represent. It allows classical statistical inference as well as machine learning inspired predictive modeling. GPPM offers frequentist and Bayesian inference without the need to resort to Markov chain Monte Carlo-based approximations, which makes the approach exact and fast. GPPMs are defined using the kernel-language, which can express many traditional modeling approaches for longitudinal data, such as linear structural equation models, multilevel models, or state-space models but also various commonly used machine learning approaches. As a result, GPPM is uniquely able to represent hybrid models combining traditional parametric longitudinal models and nonparametric machine learning models. In the present paper, we introduce GPPM and illustrate its utility through theoretical arguments as well as simulated and empirical data.

## 1. Introduction

Longitudinal data are crucial for addressing various psychological research questions, including questions related to child development, aging, and intervention research. In this paper, we focus on the analysis of (longitudinal) panel data, which we define as data that contain measurements of one or more variables from multiple individuals, each measured at multiple time points. Based on this rather broad definition, panel data encompass intensive longitudinal data, which are characterized by a relatively large number of measurements from few individuals (e.g., Walls and Schafer, 2006), as well as traditional panel data sets, which are characterized by few measurements from a relatively large number of individuals (e.g., Hsiao, 2014).

In psychological research, panel data are commonly analyzed using the general linear model (Cohen, 1968), multilevel modeling (Raudenbush and Bryk, 2001), or structural equation modeling (Bollen, 1989). Such approaches have the advantage that specification, inference, and interpretation are straightforward and well understood. However, these benefits come at the price that only relatively simple models can be expressed. Often, for example, the assumption of linear relationships between all variables is central. In addition, traditional modeling approaches are almost focused on explanatory data analysis (Shmueli, 2010; Yarkoni and Westfall, 2017). The main goal of explanatory data analysis is to estimate (parameters of) the probability distribution that generated the data, and thus, to recover relationships that hold in the population, although these relationships do not necessarily have to be causal. To this end, a model is formulated and assumed to be correctly specified, that is, to contain the population distribution. Consequently, the statistical conclusions drawn from an explanatory analysis (e.g., standard errors, *p*-values, confidence intervals) are only guaranteed to be valid if the chosen model is correct, which arguably is often not the case when analyzing panel data (e.g., Ghisletta et al., 2019).

In contrast, machine learning, with its underlying inference framework of statistical learning, changes the goal of the analysis to quantify how well a certain model predicts. Prediction may be a valuable goal in itself (e.g., prediction of treatment success or risk of developing a disease) but, also, prediction may help to generate or improve explanatory models, e.g., by providing a reference model that a purely theory-driven model has to compete with (in terms of predictive accuracy) or by providing information as to where in the input space a theory-driven model is making unsatisfying predictions (Brandmaier et al., 2016). This shift toward predictive modeling enables relatively complex models, and inferences based on the statistical learning (SL) framework do not require correctness of the model (Breiman, 2001). For example, the standard method for classification in psychology is linear logistic regression whereas in statistical learning support vector machines with a Gaussian radial basis function kernel (Vapnik, 1998) are often used, which, in contrast to linear logistic regression, allows for nonparametric models including interactions and higher-order relationships of outcomes and predictors. Inferences about the generalization performance based on statistical learning from these relatively complex models are also valid when the model is not correctly specified. As a matter of fact, in machine learning, models are often misspecified on purpose to obtain better predictions. This idea becomes particularly evident in regularization, in which parameter estimates are biased (shrunken toward zero away from their unbiased estimates) to decrease the variance of the estimates, which ultimately can lead to improved predictive accuracy (cf. the bias-variance tradeoff, see (Yarkoni and Westfall, 2017) for a detailed description).

One statistical learning method that recently has been promoted as a useful analysis tool in psychological research is Gaussian process regression (GPR) (Schulz et al., 2018). Additionally, many publications (e.g., Brahim-Belhouari and Bermak, 2004; Saatçi et al., 2010; Turner, 2012; Roberts et al., 2013) demonstrate the utility of GPR for analyzing time-series data. However, GPR cannot be easily used for the analysis of panel data. The reason is that there are currently no means to accommodate the nested nature of the data (typically, time points within persons). In this article, we extend GPR to allow for the analysis of panel data and call the resulting method Gaussian process panel modeling (GPPM). To this end, we extend GPR such that both a within-person model and a between-person model can be specified. We adapt the statistical learning inference methods used in GPR for the resulting class of GPPM models, which provides us with methods for model selection, methods to obtain person-specific predictive distributions, and methods for model validation. We provide an implementation of GPPM in form of the R (R Core Team, 2018) package “gppm” (Karch, 2018).

Although we strongly believe psychological research can profit from incorporating ideas from statistical learning (see also Brandmaier et al., 2016; Yarkoni and Westfall, 2017), the GPPM approach proposed in the present paper can also be used for explanatory data analysis. By expanding the class of possible models, GPPM may be equally beneficial for explanatory data analysis, because the ability of GPPM to specify a broad set of models might increase chances to specify a correct model. However, given its roots in statistical learning, frequentist inference procedures for GPPM—most notably hypothesis testing and confidence interval estimators for model parameters but also methods for model selection—have not yet been developed. To close this gap, we develop the standard frequentist inference procedures for GPPM in the present paper. As a result, GPPM may be conceived as a hybrid of a statistical learning and an explanatory approach that allows inference using both frameworks. Importantly, in contrast to the statistical learning conclusions, explanatory conclusions drawn from GPPM are not robust to misspecification.

GPPM is based on the so-called kernel-language for model specification. Kernels are functions that generate model-based covariances of pairs of measurements in continuous time and will be explained in more detail later on. The kernel-language builds on the concepts of the Mercer kernel (Rasmussen and Williams, 2006), which is used by many statistical learning methods such as GPR or support vector machines (Vapnik, 1998). From the perspective of longitudinal modeling, the kernel-language represents a new approach for specifying a within-person model, and thus complements the two existing approach (see, Ram and Grimm, 2015, for an overview): mathematical functions, as used in multilevel models and structural equation models, and differential equations, as used in state-space models. Importantly, the kernel-language can represent models that are not representable by either of the two existing approaches, most notably flexible nonparametric models, as typically used in machine learning. However, the kernel-language is also able to represent traditional model classes such as (linear Gaussian) structural equation models or (linear) multilevel models. Additionally, a specific strength of the kernel-language is the ability to combine models by standard combination operators easily. Consequently, GPPM enables the researcher to employ models typically used in statistical learning, models that are commonly used in psychological research, as well as a combination of the former two. We will place a particular emphasis on the utility of these combined, hybrid models, which GPPM is uniquely able to represent.

There have been previous efforts to extend GPR for *N* > 1 data. Cox et al. (2012) have adapted GPR for the analysis of computer mouse trajectories, which are nested within participants, which again are nested within conditions. Thus, these data can be considered an example of nested longitudinal data and consequently, their work as an example of using GPR for analyzing longitudinal data. However, Cox et al. (2012) tailored GPR to their specific analysis problem, whereas we aim at giving a broader perspective on GPR as a general method for panel data analysis. In addition, GPPM and the approach proposed by Cox differ with regard to model specification, estimation, and model selection. While Hall et al. (2008) did not discuss how to extend GPR for *N* > 1, they used Gaussian processes as a mathematical tool to implement a functional analysis method for panel data. Given this entirely different focus, their method is very different from GPPM, as introduced in this paper. Chen and Zhang (2019) introduced an alternative model that utilizes GPR for the analysis of (intensive) longitudinal data. Like in the dissertation monograph (Karch, 2016) and the preprint on GPPM (Karch et al., 2017), their method describes the within-person model using GPR. However, GPPM differs from their approach in terms of recommendations for model specification, implementation, and scope of possible models. Additionally, Chen and Zhang (2019) focus only on explanatory modeling and do not discuss which kind of between-person models can be implemented.

In particular, the unique contributions of this work are as follows. First, we extend GPR by a between-person model and discuss various types of between-person models that can be chosen from. Second, we elaborate on frequentist as well as statistical learning inference procedures for GPPM. Third, we relate existing approaches to model panel data in psychological research to GPPM. We show that many traditional methods, such as linear structural equation models or linear state-space models can be considered as special cases of GPPM. Fourth, an important consequence of the latter point are novel models for the analysis of panel data; in particular, the hierarchical version of models typically used in machine learning, as well as hybrid models that consist of a combination of a parametric (theory-based) model and a nonparametric statistical learning (data-driven) model. We demonstrate that these models provide advantages from a predictive (they can outperform existing models in terms of predictive accuracy) as well as explanatory (they can have a higher Bayesian posterior model probability than existing models) perspective.

The remainder of this paper is structured as follows. In the next section, section 2, we recapitulate the statistical learning method GPR as Bayesian nonparametric regression approach. In section 3, we introduce GPPM, our extension of GPR models for the analysis of panel data. In this section, we also discuss the relationship of the GPPM model class with other modeling classes; specifically, we show that both linear Gaussian structural equation modeling and linear state-space models are subsets of the GPPM model class. In section 4, we develop frequentist inference procedures, such as hypothesis testing and confidence interval estimators, for the GPPM model class. In section 5 we adapt the statistical learning inference procedures from GPR to GPPMs. In section 6, we illustrate the use of GPPM based on both simulated and a real panel data set in which participants' stance toward authoritarianism was modeled. In our demonstration, we focus on the utility of hybrid models of parametric and nonparametric kernels that GPPM is uniquely able to represent. We close with a discussion and conclusion section.

## 2. Gaussian Process Regression

### 2.1. Introduction

In this section, we briefly review GPR, which is an established statistical learning method. For an in-depth treatment, see Rasmussen and Williams (2006) and for a tutorial introduction aimed at psychologists, see Schulz et al. (2018). GPR is based on multiple linear regression. In multiple regression, the goal is to find a regression function of the form

$$\begin{array}{l} f:X\to \mathbb{R},\;f\left(x\right)=x^{⊤}b,\;Y\left(x\right)=f\left(x\right)+\epsilon ,\;\epsilon \sim N\left(0,\sigma_{\epsilon }^{2}\right). \end{array}$$

The input vector $x\in X\subseteq {\mathbb{R}}^{p}$ contains *p* predictors and the parameter vector *b* ∈ ℝ^*p*^ *p* corresponding parameters. We assume that the input vector always contains a constant predictor such that an explicit intercept is not needed. The outcome variable *Y*(*x*) represents the to-be-predicted quantity, which is assumed to vary across the predictions *f*(*x*) according to a Gaussian random variable ϵ with error variance $\sigma_{\epsilon }^{2}$. The distribution for the outcome variable *Y*(*x*) implied by its input vector *x* is thus $Y\left(x\right)\sim N\left(f\left(x\right),\sigma_{\epsilon }^{2}\right)$; and for any two input vectors *x, x*′ with *x* ≠ *x*′, Cov(*Y*(*x*), *Y*(*x*′)) = 0.

The first step toward GPR is to extend the linear regression model such that it allows for nonlinear relationships between the input vector *x* and the outcome variable (OV). This is achieved by the introduction of a function ϕ(*x*) that maps the input vector *x* into a new space, which changes the regression function to

$$\begin{array}{l} f\left(x\right)=\phi {\left(x\right)}^{⊤}b. \end{array}$$

The second step toward GPR is to employ Bayesian inference. A prior distribution is introduced for the parameters. A prior is only imposed on the coefficient vector *b* and is assumed to be Gaussian: $b\sim N\left(\mu_{b},\Sigma_{b}\right)$. The error variance $\sigma_{\epsilon }^{2}$ is assumed to be a fixed quantity, which is considered to be part of the model and thus estimated as part of the model selection procedure (see section 2.3).

The third step toward GPR is to describe the prior directly at the level of the regression function. Since every value *v* of the coefficient vector *b* translates to one particular regression function via the equation *f*(*x*|*b* = *v*) = ϕ(*x*)^⊤^*v*, imposing a prior on the coefficient vector *b* implies a prior over regression functions. Specifically, for a matrix *X* = [*x*_1_, …, *x*_*N*_], containing input vectors as columns, the prior implied for the corresponding values of the regression functions $f\left(X\right)={\left[f\left(x_{1}\right),\ldots ,f\left(x_{n}\right)\right]}^{⊤}$ can be compactly described using the matrix of transformed input vectors ϕ(*X*) = [ϕ(*x*_1_), …, ϕ(*x*_*N*_)] as follows

$$\begin{array}{l} f\left(X\right)=\phi {\left(X\right)}^{⊤}b\sim N\left(\phi {\left(X\right)}^{⊤}\mu_{b},\phi {\left(X\right)}^{⊤}\Sigma_{b}\phi \left(X\right)\right). \end{array}$$

Thus, the prior implied for the predictions of the regression function *f*(*x*) at a finite set of input vectors *X* can be described directly using a Gaussian distribution.

However, typically, the set of possible input vectors X∋x is of infinite size (e.g., time is generally considered infinite). To fully describe the prior on the level of the regression functions, the distribution of the infinite set {f(x):x∈X} has to be appropriately represented. This set is not a random vector because it is of infinite size and, consequently, its prior distribution cannot be described using a Gaussian distribution. Thus, we need to operate with an infinite-sized generalization of a random vector, which is called a stochastic process.

DEFINITION 1 (Gaussian Process). *Let* (Ω,F,ℙ) *be a probability space and (S*, Σ) *a measurable space, a stochastic process is a set of S-valued random variables on the probability space* (Ω,F,ℙ). *It can be written as* {f(x):x∈X} *using an index set* X.

A Gaussian process *is a stochastic process for which any finite subset of* {f(x):x∈X} *(which is a random vector) is distributed according to a Gaussian distribution*.

Thus, to completely describe the prior over regression function, the distribution for the Gaussian process {f(x):x∈X} needs to be specified. Just like for Gaussian random vectors, the distribution of a Gaussian process can be completely described by its first and second-order statistics. While for Gaussian random vectors, a mean vector and a covariance matrix are used, for Gaussian processes, their infinitely sized equivalents are employed; the mean function and the (Mercer) kernel.

DEFINITION 2 (Mean Function and Kernel). *Let* {f(x):x∈X} *be a stochastic process, and* $x,x^{\prime }\in X$, *then m*(*x*): = 𝔼(*f*(*x*)) *is called the mean function and k*(*x, x*′): = Cov(*f*(*x*), *f*(*x*′)) *the kernel of the stochastic process*.

The implied mean function and kernel for the Gaussian process representing the prior over regression functions are *m*(*x*) = ϕ(*x*)μ_*b*_ and $k\left(x,x^{\prime }\right)=\phi {\left(x\right)}^{⊤}\Sigma_{b}\phi \left(x^{\prime }\right)$. Thus, the choice of the transformation function ϕ(*x*) and the prior for the coefficient vector *b* determine the mean function and the kernel. For example, using the identity as transformation function and the regularizing prior $b\sim N\left(0,I\sigma_{b}^{2}\right)$, where *I* is the appropriately sized identity matrix, results in mean function *m*(*x*) = 0 and kernel $k\left(x,x^{\prime }\right)=x^{⊤}\sigma_{b}^{2}x^{\prime }$. This is the Bayesian equivalent of ridge regression (Hastie et al., 2009, Chapter 3.4.1).

The Gaussian process prior on the regression function *f*(*x*) also implies a prior on the outcome variable *Y*(*x*). Since the regression function *f*(*x*) is related to the outcome variable *Y* by the measurement equation *Y*(*x*) = *f*(*x*) + ϵ, the prior implied for the outcome variable *Y*(*x*) is a Gaussian process with mean function $m\left(x\right)=\phi {\left(x\right)}^{⊤}\mu_{b}$ and kernel $k_{y}\left(x,x^{\prime }\right)=\phi {\left(x\right)}^{⊤}\Sigma_{b}\phi \left(x^{\prime }\right)+\delta \left(x-x^{\prime }\right)\sigma_{\epsilon }^{2}$, where δ(·) is the Dirac delta function, that is, it is 0 everywhere, except at 0. We will abbreviate this as

(1) $$\begin{array}{l} Y\left(x\right)\sim \mathrm{GP}\left(\phi {\left(x\right)}^{⊤}\mu_{b},\phi {\left(x\right)}^{⊤}\Sigma_{b}\phi \left(x\right)+\delta \left(x-x^{\prime }\right)\sigma_{\epsilon }^{2}\right). \end{array}$$

Thus, the GPR model for the outcome variable *Y*(*x*) can be fully described by a mean function and a kernel. We will refer to kernels including the measurement error as *k*_*y*_ and kernels without the measurement error as *k* in the remainder of the manuscript. Model specification in GPR thus consists of choosing a mean function and a kernel. For example, the mean function and the kernel representing Bayesian linear regression with a regularizing prior and Gaussian measurement error are $m\left(x\right)=0,k_{y}\left(x,x^{\prime }\right)=x^{⊤}\sigma_{b}^{2}x^{\prime }+\delta \left(x-x^{\prime }\right)\sigma_{\epsilon }^{2}$.

### 2.2. Inference

Inference in GPR is traditionally focused on optimal predictions. To obtain unbiased estimates of the predictive accuracy, data is split into a training and a test set. The training set *D* = {(*x*_*i*_, *y*_*i*_):*i* ∈ 1, …, *N*_1_} = (*X, y*) is used to fit the model, and the goal is to obtain optimal predictions when using input vectors x^*^ that have not been in the training set. We denote such inputs vectors in a test set as the matrix $X^{*}=\left[x_{1}^{*},\ldots ,x_{N_{2}}^{*}\right]$. Bayesian statistical learning procedures typically first obtain parameter estimates in the form of the posterior distribution and then link the posterior distribution with the likelihood to obtain predictions in the form of the predictive distribution (e.g., Bishop, 2006, section 3.3). In contrast, in GPR, the predictive distribution is directly obtained in a single step.

The predictive distribution is the distribution given the model and the training set for the test set predictions, that is, *f*(X^*^)|*D*. For notational convenience, we introduce the following:

$$\begin{array}{l} \;M(X)\;=\left[\begin{array}{c} m\left(x_{1}\right)\\ m\left(x_{2}\right)\\ \vdots \\ m\left(x_{N1}\right) \end{array}\right],\\ K\left(X,X^{*}\right)=\left[\begin{array}{cccc} k(x_{1},\;x_{1}^{*}) & k(x_{1},\;x_{2}^{*}) & \ldots & k(x_{1},\;x_{N2}^{*})\\ k(x_{2},\;x_{1}^{*}) & k(x_{2},\;x_{2}^{*}) & \; & \vdots \\ \vdots & \; & \ddots & \;\\ k(x_{N}{\;}_{1},\;x_{1}^{*}) & \ldots & \; & k(x_{N}{\;}_{1},\;x_{N2}^{*}). \end{array}\right] \end{array}$$

This allows expressing the joint distribution of observations *Y*(*X*) and predictions *f*(X^*^) as follows:

$$\begin{array}{l} \left[\begin{array}{c} Y\left(X\right)\\ f\left(X^{*}\right) \end{array}\right]\sim N\left(M\left(\left[\begin{array}{c} X\\ X^{*} \end{array}\right]\right)\left[\begin{array}{cc} K\left(X,X\right)+I\sigma_{\epsilon }^{2} & K\left(X,X^{*}\right)\\ K\left(X^{*},X\right) & K\left(X^{*},X^{*}\right) \end{array}\right]\right). \end{array}$$

The predictive distribution is obtained by conditioning on the observations *y*. It has an analytical solution, which is:

(2) $$\begin{array}{l} f\left(X^{*}\right)|D\sim N\left(𝔼\left(f\left(X^{*}\right)|D\right),\mathrm{Cov}\left(f\left(X^{*}\right)|D\right)\right)\text{, with} \end{array}$$

(3) $$\begin{array}{l} 𝔼\left(f\left(X^{*}\right)|D\right)=M\left(X^{*}\right)+K\left(X^{*},X\right){\left[K\left(X,X\right)+\sigma_{\epsilon }^{2}I\right]}^{-1}\left(y-M\left(X\right)\right) \end{array}$$

(4) $$\begin{array}{l} \mathrm{Cov}\left(f\left(X^{*}\right)|D\right)=K\left(X^{*},X^{*}\right)-K\left(X^{*},X\right){\left[K\left(X,X\right)+\sigma_{\epsilon }^{2}I\right]}^{-1}K\left(X,X^{*}\right). \end{array}$$

### 2.3. Model Selection

Before obtaining predictions based on a model, the model is chosen from a set of candidate models. The model in GPR is represented by a prior over regression functions, specified by a mean function and a kernel. Thus, model selection in GPR is formally equivalent to choosing a prior.

In GPR, the prior is typically obtained using the empirical Bayes approach (Rasmussen and Williams, 2006, Chapter 5). This means the prior is chosen based on the training data *D*. For GPR, this is typically done in two ways. One approach is to choose the prior that optimizes the model evidence. This approach is well in line with GPR being a Bayesian method. The other approach is to choose the prior that optimizes the predictive accuracy as measured by cross-validation. This is well in line with GPR being a statistical learning method. However, exactly when which approach should be preferred is still discussed in the literature (Bishop, 2006; Piironen and Vehtari, 2017; Gronau and Wagenmakers, 2019, section 3.4).

Both approaches start with a set of models as represented by a parameterized mean function *m*(*x*; θ) and a parameterized kernel $k_{y}\left(x,x^{\prime };\theta \right)$. The parameters θ are so-called hyper-parameters, because every parameter value corresponds to a model. In both approaches, the hyper-parameters $\hat{\theta }$ are chosen that optimize an objective function given the training data *D* = {(*x*_*i*_, *y*_*i*_):*i* ∈ 1, …, *N*_1_} = (*X, y*) with respect to the hyper-parameters θ.

When using the model evidence approach, the objective function is the model evidence, that is, the likelihood of the training data given the model, which we denote as *p*(*y*|*X*, θ). While the model evidence can only by approximated for many models (Bishop, 2006; Kruschke, 2014), it can be computed analytically for GPR models. It corresponds to a simple evaluation of the Gaussian likelihood:

$$\begin{array}{l} p\left(y|X,\theta \right)=N\left(y;M\left(X;\theta \right),K_{y}\left(X,X;\theta \right)\right). \end{array}$$

When using the cross-validation approach for choosing a model, the objective function is the *k*-fold cross-validated prediction performance estimate (Kohavi, 1995). For a cross-validation procedure, a loss function has to be chosen, which quantifies the loss of predicting a value $\hat{y_{i}}$ when the true value is *y*_*i*_. For GPR models, which return a predictive distribution, the negative log predictive probability loss is a natural loss function (Rasmussen and Williams, 2006, p. 112) as it also takes the uncertainty of the predictions into account. The negative log predictive probability of a vector of true values $y^{*}=\left[y_{1},\ldots ,y_{N_{2}}\right]$ under the predictive distribution is

$$\begin{array}{l} -\log\left(N\left(y^{*};𝔼\left[f\left(X^{*}\right)|D\right],\mathrm{Cov}\left[f\left(X^{*}\right)|D\right]\right)\right), \end{array}$$

with the predictive mean *E*[*f*(X^*^)|*D*] and the predictive covariance matrix Cov[*f*(X^*^)|*D*]) being as defined in Equations (3) and (4) respectively. Consequently, the higher the likelihood of the true values y^*^ under the model, the lower its negative log predictive probability.

Usually, model selection approaches in both statistical learning, and inferential analyses are used to select between a finite set of candidate models. In contrast, in GPR, they are used to select between a typically infinite number of candidate models as represented by hyper-parameters from an uncountable hyper-parameter space Θ ∋ θ. To this end, iterative optimization algorithms based on the gradient of the objective function are employed. They find the best model by optimizing the chosen objective function. This model is then used to obtain predictions, as described in the previous section.

## 3. Gaussian Process Panel Models

In this section we generalize GPR to GPPM by introducing a between-person model, before introducing frequentist and statistical learning inference procedures in sections 4 and 5. To facilitate the discussion in these sections, we offer a short reinterpretation of a GPR model in the following.

### 3.1. Reinterpreting a Set of Priors as a Statistical Model

Understanding how a GPR model can be extended by a between-person model as well as complementing GPR with frequentist inference procedures is facilitated by reinterpreting the set of priors represented by a parameterized mean function *m*(*x*; θ) and kernel $k_{y}\left(x,x^{\prime };\theta \right)$ pair as a statistical model.

Each hyper-parameter value θ describes the distribution of the Gaussian process *Y*(*x*). In GPR, this distribution is interpreted to represent a prior and thus one model. Consequently, the set of distributions implied by the hyperparameter space Θ is interpreted as a set of models. An alternative interpretation is that each of these distributions is a candidate distribution for the Gaussian process *Y*(*x*). It follows that now the set of distributions implied by the parameter space Θ is one model, which in the previous interpretation was a set of models. In notation, we write

$$\begin{array}{l} Y\left(x\right)\sim \left\{GP\left(m\left(x;\theta \right),k_{y}\left(x,x^{\prime };\theta \right)\right):\theta \in \Theta \right\}, \end{array}$$

which reads: It is assumed that there exists one parameter value θ^*^ ∈ Θ such that the true mean function and kernel for the Gaussian process *Y*(*x*) are *m*(*x*; θ^*^) and *k*(*x, x*′; θ^*^).

### 3.2. Introducing Between-Person Models

Let us assume that in a longitudinal data set, *N* ∈ ℕ time series are observed. Each time series $y_{i}\in {\mathbb{R}}^{J_{i}}$ originates from one person *i* and contains *J*_*i*_ observations. With *y*_*ij*_ ∈ ℝ, we describe the *j*-th observation of person *i*. In analogy to GPR, we assume that each observation is accompanied by a corresponding input vector $x_{ij}\in X\subseteq {\mathbb{R}}^{p}$, which is also observed. As for GPR, $x,x^{\prime }\in X$ describe two arbitrary input vectors. In the simplest case, the input vector can just contain the time point of the observation (e.g., days elapsed since inception of the study), but in principle, any variable is allowed to be a member of the input vector.

For modeling, we assume that for each person their time series *y*_*i*_ is a realization^1^ of a stochastic process. Note that this assumption is reasonably general and encompasses virtually all available probabilistic methods for analyzing longitudinal data, including advanced methods such as nonlinear state-space models (Chow and Zhang, 2013).

The reinterpretation of GPR models is in line with this formalism. Essentially, with GPR, a model for the stochastic process of a single person can be defined assuming the stochastic process is a Gaussian process. Then, each person's time series *y*_*i*_ is considered a realization of a Gaussian process *Y*_*i*_(*x*) with true distribution as follows

$$\begin{array}{l} Y_{i}\left(x\right)\sim GP\left(m_{i}^{*}\left(x^{\prime }\right),k_{i}^{*}\left(x,x^{\prime }\right)\right). \end{array}$$

The mean function $m_{i}^{*}\left(x\right)$ and the kernel $k_{i}^{*}\left(x,x^{\prime }\right)$ represent the true distribution of the person-specific Gaussian process. Thus, for each person, a model can be formalized using a parameterized mean function and kernel.

However, so far, it is not possible to specify relationships between persons, that is, a between-person model. The most straightforward approach to specify a between-person model is to assume that each person's time series is a realization of the same Gaussian process and that the person-specific Gaussian processes are mutually independent. The statistical model implied by this approach is

(5) $$\begin{array}{r} Y_{i}\left(x\right)\sim \left\{GP\left(m\left(x;\theta \right),k_{y}\left(x,x^{\prime };\theta \right)\right):\theta \in \Theta \right\} \end{array}$$

for every person. We call such a model a Gaussian process panel model (GPPM). Equivalently to GPR models, GPPMs are specified by choosing the predictors, the mean function, and the kernel.

Although the mean function and the kernel are assumed to be identical for each person, this does not imply that there are no between-person differences. Many forms of between-person differences can be specified using this formalism. More specifically, the model displayed in Equation (5) does not necessarily assume that the *true* distribution of the person-specific Gaussian processes is the same for each person. We will discuss this in more detail in the following section.

### 3.3. Supported Between-Person Models

Let θ_*ip*_ be the person-specific variant of parameter θ_*p*_. If the person-specific parameter θ_*ip*_ is considered a realization of a between-person probability distribution ℙ(θ), then we speak of probabilistic between-person model. If, in contrast, the person-specific parameter θ_*ip*_ is determined by a function *f*(τ_*i*_, θ_τ_) = θ_*ip*_ with a vector of time-invariant covariates *t*_*i*_ and a parameter vector θ_τ_ as input then we speak of a deterministic between-person model. Note that this formalism also covers conditional distributions ℙ(θ|τ_*i*_), as this can be achieved by combining deterministic and probabilistic models.

In GPPM, a Gaussian between-person distributions for linear mean parameters can be implemented by simply modifying the mean function and kernel. Let the mean function be of the form

(6) $$\begin{array}{r} m\left(x;\theta \right)=f{\left(x;\theta_{1}\right)}^{⊤}\theta_{2}+h\left(x;\theta_{3}\right), \end{array}$$

where the parameter vector θ = [θ_1_, θ_2_, θ_3_] is partitioned into parameter vectors θ_1_, θ_2_, θ_3_. *f*(θ_1_) is a vector-valued, and *h*(θ_3_) a scalar-valued function. The parameters in the vector θ_2_ are what refer to as linear mean parameters. A probabilistic between-person model is introduced by individualizing the linear parameters in the vector θ_2_ and assuming that the corresponding individualized parameter has the between-person distribution $\theta_{i2}\sim N\left(\mu_{\theta_{2}},\Sigma_{\theta_{2}}\right)$. As a result, the mean function itself becomes a Gaussian process with mean function and kernel

$$\begin{array}{l} \;𝔼\left[m\left(x;\theta \right)\right]=𝔼\left[f{\left(x;\theta_{1}\right)}^{⊤}\theta_{i2}+h\left(x;\theta_{3}\right)\right]\\ \;=f{\left(x;\theta_{1}\right)}^{⊤}\mu_{\theta_{2}}+h\left(x;\theta_{3}\right)\\ \mathrm{Cov}\left(m\left(x;\theta \right),m\left(x^{\prime };\theta \right)\right)=f{\left(x;\theta_{1}\right)}^{⊤}\Sigma_{\theta_{2}}f\left(x^{\prime };\theta_{1}\right) \end{array}$$

Consequently, the resulting GPPM is

(7) $$\begin{array}{l} \begin{array}{c} \;\tilde{m}\left(x;\theta \right)=f\left(x;\theta_{1}\right)\mu_{\theta_{2}}+h\left(x;\theta_{3}\right)\\ \tilde{k}\left(x,x^{\prime };\theta \right)=k\left(x,x^{\prime };\theta \right)+f{\left(x;\theta_{1}\right)}^{⊤}\Sigma_{\theta_{2}}f\left(x^{\prime };\theta_{1}\right) \end{array} \end{array}$$

To investigate whether other types of between-person models besides a Gaussian between-person model on mean parameters can be defined, we use the mathematical equivalence of between-person models and priors. Introducing a between-person distribution for a given parameter is equivalent to introducing a prior distribution over that parameter, even though either approach may have quite different implications in practice. With this equivalence in mind, we may then regard the original mean function and kernel as the likelihood. To express the new statistical model, the marginal likelihood has to be obtained, that is, a weighted average of the likelihood using the prior as weighing function. We showed that for a Gaussian likelihood and a Gaussian prior on linear parameters of the mean, the marginal likelihood is again Gaussian (also see Bishop, 2006, Chapter 2.3.3). Between-person distributions other than the Gaussian will typically not lead to the marginal likelihood being Gaussian. The Gaussian prior is the only commonly used prior that has this property. The same is true for between-person distributions on the kernel parameters, as for most commonly used priors on variance parameters, the resulting marginal likelihood is not a Gaussian. To implement non-Gaussian between-person distributions and between-person distributions of variance parameters in the GPPM framework, an extension of the basic GPPM formalism is needed, which remains subject to future research.

Deterministic between-person models, that is, parameter heterogeneity that is governed by a deterministic function *f*(τ_*i*_, θ_τ_) = θ_*ip*_, can be implemented by changing the mean function, the kernel, and the predictors in the input vector *x*. For this, we use a method that is able to implement parameter heterogeneity for every single observation: Let θ_*p*_ be a parameter of the mean function for which a person- and potentially observation-specific variant θ_*ijp*_, determined by a function *f*(τ_*ij*_, θ_τ_) = θ_*ijp*_, is desired. We now assume that the vector τ also contain time-varying and time-invariant covariates. To implement this, one simply needs to replace the parameter θ_*p*_ by *f*(*t*_*ij*_, θ_τ_) in the mean function, add the time-variant and time-invariant covariates in the vector τ_*ij*_ to the input vector *x*, and add the parameter vector θ_τ_ to the parameter vector θ.

The same concept can be used to implement person- and observation specific heterogeneity for parameters in the kernel. When implementing observation-specific heterogeneity, it is important to note that a kernel describes the model for the pairwise covariance between all observations. Thus, observation-specific heterogeneity needs to take the state values of two observations into account. This however, can be easily accommodated for by letting the function governing the parameter heterogeneity depend on a pair of covariate vectors τ_*ij*_, τ_*ik*_, that is, it changes to *f*(τ_*ij*_, τ_*ik*_, θ_τ_). Besides that small change, everything works analogously for mean functions.

### 3.4. Model Specification and Relation to Other Model Families

Given a panel data set, a GPPM for an outcome variable *Y* is defined by identifying predictor variables in the input vector, which at least includes the time of measurement, and by specifying a mean function and a kernel. Model specification is facilitated by the fact that models can be specified by simple combination rules. For example, a valid mean function can be created by summation of, multiplication of, and scaling of base mean functions. The same operators can be used to create a new kernel based on combinations of base mean functions and kernels (see Duvenaud et al., 2013, for details). Some of these operators have straightforward interpretations. For example, the sum of two Gaussian processes with mean functions *m*_1_, *m*_2_ and kernels *k*_1_, *k*_2_ has mean function *m* = *m*_1_ + *m*_2_ and kernel *k* = *k*_1_ + *k*_2_.

With GPPM being a hybrid of a statistical learning and an explanatory method, a model can be specified using different strategies.

One approach to model specification is to translate a substantive theory into its GPPM representation. For example, a very simple theory could posit that there is no change over time and no between-person differences; that is, all observed differences across persons and measurement occasions are solely attributed to measurement error. This would translate into the following GPPM

$$\begin{array}{l} m\left(x;\theta \right)=\mu_{I},\;k_{y}\left(x,x^{\prime };\theta \right)=\delta \left(x-x^{\prime }\right)\sigma_{\epsilon }^{2}, \end{array}$$

with μ_*I*_ ∈ ℝ.

Another approach for specifying a model is to translate a traditional model into a GPPM. For example, we now translate the linear latent growth curve model (Preacher et al., 2008), which assumes that each person's trajectory over time follows a linear trend while allowing individual differences in intercept and linear slope. The only predictor needed is time. We, thus, denote the input vector with *t* instead of *x*. The GPPM representation of the univariate linear latent growth curve model is

(8) $$\begin{array}{l} \;m\left(t;\theta \right)=\underset{\text{constant}}{\underset{︸}{\mu_{I}}}+\underset{\text{linear}}{\underset{︸}{\mu_{S}t}},\\ k_{y}\left(t,t^{\prime };\theta \right)=\underset{\text{constant}}{\underset{︸}{\sigma_{I}^{2}}}+\underset{\text{linear}}{\underset{︸}{t\sigma_{S}^{2}t^{\prime }}}+\underset{\text{covariance}}{\underset{︸}{\sigma_{IS}\left(t+t^{\prime }\right)}}+\underset{\text{noise}}{\underset{︸}{\delta \left(t-t^{\prime }\right)\sigma_{\epsilon }^{2}}}, \end{array}$$

with μ_*I*_ being the mean of the intercept, μ_*S*_ the mean of the slope, $\sigma_{I}^{2}$ the variance of the intercept, $\sigma_{S}^{2}$ the variance of the slope, σ_*IS*_ the covariance between intercept and slope, and $\sigma_{\epsilon }^{2}$ the variance of the measurement error, which is assumed to be constant across time. The latent growth curve model illustrates the combination rules that are foundational to the construction of GPPM. We annotated Equation 8 with the commonly used names of the base mean functions and kernels that form the LGCM in our representation. An important advantage of translating a traditional model into a GPPM is that it can be used with a statistical learning inference approach, as discussed in more detail further below. Another advantage is that the GPPM representation is inherently a continuous-time model that allows to consider person-specific time points of measurement, irregular intervals between measurements, and naturally allows us to interpolate and extrapolate unobserved time points.

Another approach for model specification is to adapt a model typically used for GPR. Many GPR practitioners rely on a default model, which is flexible enough to approximate most (smooth) functions, known as the universal approximating property (Micchelli et al., 2006). This model is known as the squared exponential model and is defined as follows:

$$\begin{array}{l} m\left(x;\theta \right)=0,\;k\left(x,x^{\prime };\theta \right)=\sigma_{se}^{2}\exp\left(-\frac{||x-x^{\prime }|{|}^{2}}{l}\right). \end{array}$$

The parameter $\sigma_{se}^{2}$ governs the variance of the process and the strictly positive parameter *l* governs how fast the correlation drops between two variables *Y*(*x*), *Y*(*x*′) as a function of the squared euclidean distance of their input vectors *x* and *x*′. We will adapt and explore the utility of the squared exponential model for longitudinal data in the illustrations section.

Another valuable property for model specification is that the family of GPPMs subsumes many other model families such as longitudinal (linear Gaussian) structural equation models, and (linear Gaussian continuous-time) state-space models (Karch, 2016). Essentially, a linear Gaussian continuous-time state-space model describes a model for a Gaussian process via stochastic differential equations, whereas in GPPM a model for a Gaussian process is described using the mean function and the kernel. By definition, any distribution of a Gaussian process can be specified via a mean function and a kernel, whereas only a subset of distributions can be represented by stochastic differential equations. For example, the squared exponential model cannot be represented as a state-space model (Särkkä and Hartikainen, 2012). Similarly, in structural equation modeling, a model for a Gaussian random vector is specified by restricting its mean vector and covariances matrix whereas in GPPM a model is described on a Gaussian process (the generalization of a Gaussian random vector) by restricting its mean function (the generalization of a mean vector) and kernel (the generalization of a covariance matrix).

The rules for combining models, along with the fact that GPPM can represent a wide range of different models, can also be used to mix models from different traditions. In the illustration section, we will explore this idea by combining the squared exponential model typically used in statistical learning with a growth curve model.

## 4. Frequentist Inference for Gaussian Process Panel Models

### 4.1. Implied Statistical Model

Frequentist inference theory requires a statistical model, which is a set of candidate distributions for a random vector. A GPPM, as defined in Equation (5), is a set of candidate distributions for a stochastic process and thus not a proper statistical model. However, while a stochastic process is of infinite size, the observations drawn from it, in our case a panel data, set are necessarily finite. Thus, the data set can be seen as a realization of a finite-dimensional subset of the stochastic process, which is a random vector.

The statistical model implied by a GPPM is as follows. Let $X_{i}\in {\mathbb{R}}^{p\times J_{i}}$ be a matrix where each column $x_{ij}\in X\subseteq {\mathbb{R}}^{p}$ contains the input vector for the *j*-th observation of person *i*, that is, for the observation *y*_*ij*_ ∈ ℝ. The statistical model for all observations *y*_*i*_ = [*y*_1_, …, *y*_*J*_*i*__] of person *i* implied by a GPPM with mean function *m* and kernel *k*_*y*_ is

$$\begin{array}{l} p\left(y_{i}|X_{i}\right)\in \left\{N\left(y_{i};M\left(X_{i};\theta \right),K_{y}\left(X_{i},X_{i};\theta \right)\right):\theta \in \Theta \right\}. \end{array}$$

The statistical model implied for a longitudinal data set *D* = (*X, y*), with *X* = (*X*_1_, …, *X*_*N*_) and *y* = (*y*_1_, …, *y*_*N*_), follows from the mutual independence assumption and is

$$\begin{array}{l} p\left(y|X\right)\in \left\{\overset{N}{\underset{i=1}{\prod }}N\left(y_{i};M\left(X_{i};\theta \right),K_{y}\left(X_{i},X_{i};\theta \right)\right):\theta \in \Theta \right\}. \end{array}$$

This is a regular statistical model for which regular inference procedures can be derived as we will show in the following.

### 4.2. Point Estimation

Here, we show how to obtain maximum likelihood estimates for a GPPM and investigate their frequentist properties. To this end, the parameters $\hat{\theta }$ need to be found that maximize the likelihood of the data, that is,

$$\begin{array}{l} \hat{\theta }=\underset{\theta \in \Theta }{arg max}p_{\theta }\left(y|X\right) \end{array}$$

with likelihood function

(9) $$\begin{array}{r} p_{\theta }\left(y|X\right)=\overset{N}{\underset{i=1}{\prod }}N\left(y_{i};M\left(X_{i};\theta \right),K_{y}\left(X_{i},X_{i};\theta \right)\right) \end{array}$$

Equivalently, the log likelihood

$$\begin{array}{l} \log\left(p_{\theta }\left(y|X\right)\right)=\overset{N}{\underset{i=1}{\sum }}\log\left(N\left(y_{i};M\left(X_{i};\theta \right),K_{y}\left(X_{i},X_{i};\theta \right)\right)\right) \end{array}$$

can be maximized.

The maximum likelihood estimates for a GPPM can typically not be derived analytically. For this reason, we employ gradient descent algorithms as they are commonly used in, for example, structural equation modeling. The required gradient of the log likelihood function log(*p*(*y*|*X*, θ)) can be calculated analytically:

$$\begin{array}{l} \frac{\partial \log\left(p_{\theta }\left(y|D\right)\right)}{\partial \theta_{p}}=\overset{N}{\underset{i=1}{\sum }}\frac{1}{2}{\tilde{y_{i}}}^{⊤}\Sigma_{i}^{-1}\frac{\partial \Sigma_{i}}{\partial \theta_{p}}\Sigma_{i}^{-1}\tilde{y_{i}}\\ \;-\frac{1}{2}\mathrm{tr}\left(\Sigma_{i}^{-1}\frac{\partial \Sigma_{i}}{\partial \theta_{p}}\right)+\frac{\partial \mu_{i}}{\partial \theta_{p}}\Sigma_{i}^{-1}\tilde{y_{i}} \end{array}$$

where μ_*i*_(θ) = *M*(*X*_*i*_; θ) is the model-implied mean vector for person *i*, Σ_*i*_(θ) = *K*_*y*_(*X*_*i*_, *X*_*i*_; θ) the model implied covariance matrix, and $\tilde{y_{i}}\left(\theta \right)=y_{i}-M\left(X_{i};\theta \right)$ is the derivation of the observations from the model implied mean. For notational simplicity, we have decided to not explicitly state the dependence on θ for these terms.

Under certain regularity conditions (Taboga, 2012b) maximum likelihood estimates are consistent, efficient, and have asymptotically a Gaussian sampling distribution with the Fisher information matrix as covariance matrix (Taboga, 2012b). A comprehensive discussion of all regularity conditions is beyond the scope of this text. However, it is important to note that some conditions, such as the integratability of the log-likelihood function, are always met if a Gaussian statistical model is assumed. Others depend on the specific choice of mean and kernel function and may be violated.

One particular crucial model-dependent condition, which needs to be met, is identification. The mean and the kernel function must be such that each unique combination of parameter values implies one unique likelihood function (see Equation 9). Note that for GPPMs identification thus does not only depend on the mean function and kernel but also the data (empirical identification). For example, the mean function *m*(*t*; [*a, b*]) = *a* + *bt*, representing an average linear change over time, is identified if there are at least two measurement occasions but not otherwise.

Beyond identification, the possible violations are largely shared among modeling approaches. We refer the reader to the discussion in the context of structural equation modeling (e.g., Stoel et al., 2006).

We thus expect the maximum likelihood estimates for GPPMs to be consistent, efficient, and to have an asymptotically Gaussian sampling distribution for all well-behaved models.

### 4.3. Hypothesis Tests, Confidence Intervals, Model Selection, and Validation

For hypothesis tests, the likelihood ratio test with an asymptotic Chi-squared sampling distribution of the test statistic can be used (Taboga, 2012a). This follows directly from the maximum likelihood estimators being asymptotically normal.

As for structural equation modeling (Pek and Wu, 2015), two main approaches for computing confidence intervals can be used: Wald-type and likelihood-based methods. Essentially, Wald-type confidence intervals invert the Wald-test, whereas likelihood-based methods invert the likelihood-ratio test. Thus, the validity of these methods relies on the validity of their corresponding tests, which in turn follows from the maximum likelihood estimator to have an approximate Gaussian sampling distribution.

For model selection, the prototypical frequentist approach to test between two competing nested models using a hypothesis like the likelihood-ratio test can be used. Alternatively, when two models are not nested, many different approaches for selecting between two models exist. As a start, we adapt two popular, general, and simple approaches, namely, the Akaike information criterion (AIC) and the Bayesian information criterion (BIC). Both measures only depend on the log-likelihood of a model at the maximum likelihood estimate $\hat{\theta }$ and the number of parameters and thus can also be used to select between two GPPMs.

For model validation, the prototypical frequentist approach is to test the model itself using a hypothesis test. In structural equation modeling, for example, this is done by testing the model against the saturated model, that is, the model “any Gaussian distribution.” This essentially results in a comparison of the covariance matrix estimate under the model with the sample covariance matrix. When using a GPPM, the equivalent would be to test the obtained kernel under a GPPM, with a kernel estimate under the model “any kernel function.” Intuitively, estimating this kernel seems impossible without making additional assumptions, since with every new data point, also a new parameter is introduced. Whether and how this kernel can be estimated remains to be investigated.

## 5. Statistical Learning Inference for Gaussian Process Panel Models

### 5.1. Prerequisites

To develop statistical learning inference methods for GPPMs, we interpret a GPPM as a prior over all potential observations. That is, we interpret a GPPM as represented by a mean function and a kernel to describe a prior for the set of potentially infinitely many Gaussian processs (GPs) $\left\{Y_{i}\left(x\right):i\in I,x\in X\right\}$ with *I* containing the indices for all, potentially infinitely many, persons and X denoting the set of possible input vectors. Since in a GPPM independence and identical distribution is assumed between the person-specific GPs, it is sufficient to specify one shared mean function and kernel. The interpretation of a GPPM as representing a set of priors is in line with the interpretations used in GPR. Consequently, the statistical learning procedures used for GPR can be applied to GPPMs with only slight adaptations.

Statistical learning is primarily concerned with estimating generalization error to make decisions about which model is the best-fitting model. To obtain unbiased estimates of this generalization error, one way is to use independent training and test sets. The training set is used for model fitting and the test set for model evaluation. The standard approach to obtain training and a test set is to split the data set. A common approach is random splitting, assuming independence of all observed cases. In the context of digit classification, for example, *x*_*i*_ is a matrix of brightness values for an image and *y*_*i*_ the corresponding digit for that image. Neither the brightness values *x*_*i*_ nor the digit label *y*_*i*_ of one particular image typically contains information about any other image.

For longitudinal data, the situation is more complex. First, the data set is inherently nested. There are different persons, and each person has been observed at multiple measurement occasions. Mathematically, we denote the *j*-th observation of person *i* as (*x*_*ij*_, *y*_*ij*_). Thus, when splitting a longitudinal data set, the first crucial question is whether to split based on persons, measurement occasions, or both. As we will show in the next section, GPPM can obtain predictions for all these scenarios. When splitting based on persons, all observations (*x*_*ij*_, *y*_*ij*_) for which the person index *i* is greater than some threshold could be put in the test set and all others in the training set. Importantly, the data of each person is either entirely in the training or in the test set. In a similar fashion, when splitting a longitudinal data set based on measurement occasions, one could distribute all observations earlier than a given time point to the training set and all remaining measurements to the test set.

How to split the data is guided by which generalization performance of the model is of core interest. When the ability of the model to predict observations for persons who are not in the training set is of interest, the appropriate split is by persons. In contrast, if the ability of the model to predict observations in the data set for future measurement occasions that are not in the training set is of interest, the appropriate split is by measurement occasions.

When splitting by measurement occasions, special care has to be taken, because the common assumption that observations in the training and test set are independent, can be easily violated. This problem is extensively discussed in the time series literature, and we refer the interested reader to Bergmeir and Benítez (2012).

### 5.2. Prediction

The procedure of how to obtain predictions for data not in the training set follows closely the idea underlying GPR. The joint distribution of the training data and the test data is implied by the model and then conditioned on the training observations *y*. This process can be simplified by the following observation: As a result of the independence between persons assumption, the predictions for a particular person are only influenced by observations from the same person, and the predictive distributions for different persons are independent of each other. Thus, the predictive distribution can be calculated independently for each person *i* in the test set.

Two scenarios to obtain a predictive-distribution for a person *i* must be distinguished. First, if there are no observations from person *i* in the training data. In this case, the predictive distribution is independent of the training data. The predictive distribution for predictions of interest $Y_{i}\left(X_{i}^{*}\right)=\left[Y_{i}\left(x_{i1}^{*}\right),\ldots ,Y_{i}\left(x_{iJ_{i}^{*}}^{*}\right)\right]$ is simply:

$$\begin{array}{l} Y_{i}\left(X_{i}^{*}\right)|D\sim N\left(M\left(X_{i}^{*};\hat{\theta }\right),K_{y}\left(X_{i}^{*},X_{i}^{*};\hat{\theta }\right)\right). \end{array}$$

Second, if there are observations from person *i* in the training data, the joint distribution of observations *Y*_*i*_(*X*_*i*_) = [*Y*_*i*_(*x*_*i*1_), …, *Y*_*i*_(*x*_*i*_*J*__*i*__)] and predictions of interest $Y_{i}\left(X_{i}^{*}\right)$ has to be formulated:

(10) $$\begin{array}{r} \left[\begin{array}{c} Y_{i}\left(X_{i}\right)\\ Y_{i}\left(X_{i}^{*}\right) \end{array}\right]\sim N\left(M\left(\left[\begin{array}{c} X_{i}\\ X_{i}^{*} \end{array}\right];\hat{\theta }\right),\left[\begin{array}{cc} K\left(X_{i},X_{i};\hat{\theta }\right) & K\left(X_{i},X_{i}^{*};\hat{\theta }\right)\\ K\left(X_{i}^{*},X_{i};\hat{\theta }\right) & K\left(X_{i}^{*},X_{i}^{*};\hat{\theta }\right) \end{array}\right]\right) \end{array}$$

In complete equivalence to the predictive distribution in GPR, the predictive distribution for $Y_{i}\left(X_{i}^{*}\right)$ is obtained by conditioning on the training data *D*. As discussed before, only the observations *y*_*i*_ from person *i* are needed because $Y_{i}\left(X_{i}^{*}\right)|D=Y_{i}\left(X_{i}^{*}\right)|X_{i},y_{i}$:

(11) $$\begin{array}{l} Y_{i}(X_{i}^{*})|X_{i},\;y_{i}∼N(𝔼(Y_{i}(X_{i}^{*})|X_{i},\;X_{i}^{*},\;y_{i}),\text{ Cov}(Y_{i}(X_{i}^{*})|X_{i},\;X_{i}^{*},\;y_{i}))]\\ \;,\text{ with} \end{array}$$

(12) $$\begin{array}{l} 𝔼\left(Y_{i}\left(X_{i}^{*}\right)|X_{i},y_{i}\right)=M\left(X_{i}^{*}\right)+K\left(X_{i}^{*},X_{i}\right){\left[K\left(X_{i},X_{i}\right)\right]}^{-1}\left(y_{i}-M\left(X_{i}\right)\right) \end{array}$$

(13) $$\begin{array}{l} \mathrm{Cov}\left(Y_{i}\left(X_{i}^{*}\right)|X_{i},y_{i}\right)=K\left(X_{i}^{*},X_{i}^{*}\right)-K\left(X_{i}^{*},X_{i}\right){\left[K\left(X_{i},X_{i}\right)\right]}^{-1}K\left(X_{i},X_{i}^{*}\right). \end{array}$$

where we dropped the dependence on $\hat{\theta }$ for notational convenience.

The predictive distribution can be reduced to both an interval or a point estimate.

For point estimation, in principle, any Bayesian technique to reduce a posterior distribution to a parameter estimate can be used. However, since the predictive distribution of a GPPM is Gaussian, the two most common techniques, using the mode (maximum a posterior estimation) or expectation (minimum mean square error estimation) of the posterior, both correspond to the mean of the predictive distribution. That is, the recommended point estimate for the prediction implied by a input vector $x_{i}^{*}$ is $𝔼\left(Y_{i}\left(x_{i}^{*}\right)|X_{i},y_{i}\right)$, which a special case of Equation (12).

To obtain an interval estimate for the predictions, credible intervals can be constructed from the Gaussian predictive distribution. Since the predictive distribution is Gaussian, a credible interval can be obtained by using $𝔼\left(Y_{i}\left(x_{i}^{*}\right)|X_{i},y_{i}\right)\pm c_{\alpha }\sqrt{\mathrm{Var}\left(Y_{i}\left(x_{i}^{*}\right)|X_{i},y_{i}\right)}$. Because the variance of a variable is equivalent to the covariance with itself, $\mathrm{Var}\left(Y_{i}\left(x_{i}^{*}\right)|X_{i},y_{i}\right)$ is a special case of Equation (13). The critical value *c*_α_ has to be chosen based on the cumulative density function of the Gaussian distribution to obtain the desired credibility 1 − α.

Predictions can also be obtained for latent variables using the same framework. All that is needed is a model for the joint distribution of latent variables and observations. In the illustration section, we will demonstrate this idea.

### 5.3. Model Selection and Validation

The statistical learning approaches used in GPR for model selection and validation can be readily adapted to GPPM. Remember that for the statistical learning perspective on GPPM each hyper-parameter vector value θ of the mean function and kernel represents one prior and consequently one model.

For the model evidence maximization approach to select a model and thus a hyperparameter vector value θ, the hyperparameter vector value θ that maximizes

$$\begin{array}{l} p\left(y|X,\theta \right)=\overset{N}{\underset{i=1}{\prod }}N\left(y_{i};M\left(X_{i};\theta \right),K_{y}\left(X_{i},X_{i};\theta \right)\right). \end{array}$$

is selected. Note that this expression is identical to the likelihood function used for maximum likelihood estimation. Thus, the best model $\hat{\theta }$ from the statistical learning perspective and the maximum likelihood parameter $\hat{\theta }$ from the explanatory perspective are the same, only their interpretation differs. Also, the gradient-descent algorithm developed for maximum likelihood estimation can be re-used for model selection.

Since GPPM comes with mechanisms to obtain predictions, model selection procedures that estimate the predictive performance, most notably cross-validation, can also be employed. Because cross-validation is essentially a repeated splitting in training and validations sets, the same complications discussed earlier apply. Another issue of using cross-validation for model selection is that cross-validation estimates tend to have a high variance when using small data sets (Piironen and Vehtari, 2017). This issue can be partly resolved by repeated cross-validation, which decreases the variance but increases the computational demands. Another approach is to use the model evidence maximization approach instead, which we will consequently focus on in this paper.

To validate a selected GPPM also its predictive performance is estimated. However, the data set for performance estimation must be independent of the data set used for model selection to avoid overly optimistic estimates. If cross-validation is used for model selection and performance estimation, this leads to a process called nested cross-validation, which is described in detail in Karch et al. (2015).

## 6. Illustrations

### 6.1. Simulated Data: Utility of the Statistical Learning Perspective

Based on simulated data, we will first demonstrate how the statistical learning inference methods for GPPMs enable valid estimation of the predictive accuracy from standard restrictive longitudinal models such as the latent growth curve model (LGCM) even if the assumptions are violated. Second, we will demonstrate the utility of the more flexible models representable in GPPM. Third, and most importantly, we will showcase the ability of GPPM to express hybrid models that consist of a combination of standard restrictive models as well as flexible statistical learning models and the utility of these combinations. GPPM is uniquely equipped to express such models as it can represent the majority of restrictive longitudinal models typically used in psychology as well as a large class of statistical learning models and contains a set of easy rules to combine models.

To begin with, we start with the linear LGCM, which is one of the most frequently used model for analyzing longitudinal panel data in psychological research. It is a prototypical example of a restrictive model as it assumes that within-person change is linear. Additionally, inference is typically performed using classical frequentist inference methods, which crucially assume the correctness of the model. It is well known that this can lead to dramatically wrong conclusions if the assumptions are not met (e.g., Ghisletta et al., 2019).

To demonstrate this, we assume that an “exponential rise to the limit” is the true data generating model. This model can be interpreted to represent the typical skill development observed in training studies. The within-person model is

$$\begin{array}{l} Y_{i}\left(t\right)=b_{i}+d_{i}\exp\left(-ts\right)+\epsilon_{i}\left(t\right),\text{with }\epsilon_{i}\left(t\right)\sim GP\left(0,\sigma_{\epsilon }^{2}\right) \end{array}$$

At time *t* = 0, the model implies 𝔼(*Y*_*i*_(0)) = *b*_*i*_ + *d*_*i*_. Thus, the parameter combination *b*_*i*_ + *d*_*i*_ serves as intercept in this model. For, *t* → ∞, the model implies $\underset{t\to \infty }{\lim}𝔼\left(Y_{i}\left(t\right)\right)=b_{i}$. Consequently, the model implies that each persons skill level saturates at some point. The strictly positive parameter *s*_*i*_ represents how fast person *i* reaches their natural limit. For the between-person model, we assume $b_{i}\sim N\left(\mu_{b},\sigma_{b}^{2}\right),d_{i}\sim N\left(\mu_{d},\sigma_{d}^{2}\right)$, $s_{i}\sim N\left(\mu_{s},\sigma_{s}^{2}\right)$, and *b*_*i*_, *d*_*i*_, *s*_*i*_ being mutually independent. For *s*_*i*_, we use a truncated normal to avoid negative values.

**Figure 1:**
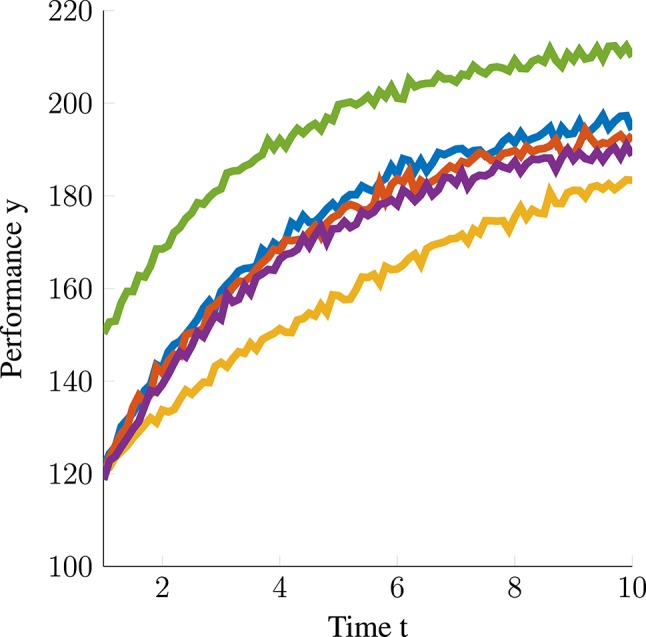
Five model-implied trajectories simulated from the “exponential rise to limit model.” Each line represents the skill trajectory of one person.

For generating data from this model, we used the following parameter values μ_*b*_ = 200, $\sigma_{b}^{2}=50$, μ_*d*_ = − 100, $\sigma_{d}^{2}=50$, μ_*s*_ =.25, $\sigma_{s}^{2}=0.01,\sigma_{\epsilon }^{2}=1$. We generated data for 200 persons with 91 measurements each; all taken at the same time points {1, 1.1, 1.2, …, 10}. Example trajectories of this model are displayed in Figure 1.

The linear LGCM or any of the typical extensions to polynomials of a higher order, such as quadratic or cubic, do not contain the data generating model and are thus misspecified.

**Figure 2:**
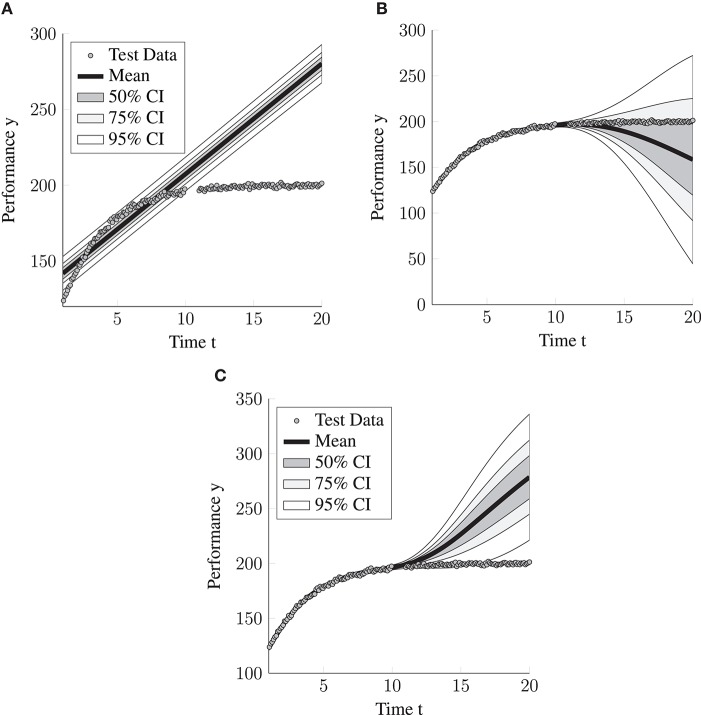
Visualization of the predictive distributions on the “exponential rise to the limit” data for the three considered models. CI = credibility interval. **(A)** LGCM. **(B)** squared exponential (SE) Model. **(C)** LGCM+SE.

In contrast, representing the LGCM as a GPPM allows performing valid inference using the LGCM on data simulated from the “exponential rise to the limit” model. The statistical learning framework applied to the LGCM first results in parameter estimates, which are equivalent to the maximum likelihood estimates. However, importantly, they are virtually ignored and only used to obtain the inferential object of interest, the predictive distribution. Using the maximum likelihood parameters $\hat{\theta }$, the predictive distribution for each person can be obtained according to Equations (10–12). We display the predictive distribution for one selected person in Figure 2A.

Whereas the predictive distributions crucially depend on the model, their performance is evaluated in a model-free fashion, which leads to their evaluation being independent of the validity of the model assumptions. For choosing the appropriate test set, it is crucial to distinguish which kind of predictive performance we want to assess. Here, we focus on assessing how well the model can make predictions for unobserved time points for persons within the sample. A second decision has to be made regarding the assessment of inter- or extrapolation capabilities. For assessing how well the model interpolates, we created a test set that contains the measurements for all persons in the original sample at time points {1.05, 1.15, …, 9.95}. For assessing how well the model extrapolates, we used time points {11, 11.1, …, 20}.

As loss function, we use the negative log predictive probability (NLPP). We report the average NLPP across persons. To make the average NLPP loss more interpretable, we normalized it using the best possible model, the Bayes-optimal model, as reference. Because we have generated data with an error variance of 1 (also referred to as “irreducible error”), the Bayes-optimal model has an expected NLPP on the test set of the size of the interpolation set of $90\cdot \log\left(\sqrt{2\pi e}\right)=127.70$, where 90 the number of measurements per person and $\log\left(\sqrt{2\pi e}\right)$ the entropy of the standard normal distribution. We subtracted this number from the estimated average NLPPs to obtain normed NLPPs.

Not surprisingly, the results shown in Table 1 reveal that the LGCM interpolates and extrapolates rather poorly. The interpolation normed NLPP was 152.1. The extrapolation NLPP was even higher at 317.77. The reason for this difference can be understood by looking at Figure 2A. In the interpolation range, the LGCM still provides a decent approximation of the nonlinear trend. However, in the extrapolation range, the LGCM confidently makes wrong predictions, which is caused by the predictions being based on incorrect, strict assumptions.

**Table 1:** Negative log predictive probabilities on the “exponential rise to the limit data” for the compared models as estimated by the different test sets.

**Model**	**Interpolation**	**Extrapolation**	**Combined**
LGCM	152.1	317.77	600.2
SE Model	2.33	11.89	13.862
LGCM+SE Model	2.21	16.86	18.853

The flexible statistical learning models representable in GPPM address this issue of the LGCM since they have specifically been designed to be able to interpolate a large set of functions well. Consequently, given enough data, they will reach an almost perfect interpolation performance for a large set of developmental trajectories. Thus, while those models are misspecified, given enough data, they predict essentially equally well as the true model. One such model is represented by the squared exponential (SE) kernel, which we have introduced earlier and repeat here:

$$\begin{array}{l} m\left(t;\theta \right)=0,\;k_{se}\left(t{,}^{\prime }t;\theta \right)=\sigma_{se}^{2}\exp\left(-\frac{t-t^{\prime }}{l}\right). \end{array}$$

The SE model represents the family of smooth predictive functions. Importantly, in regions where no data has been observed, the SE model falls back to predicting 0. Thus, it can be interpreted as regularizing toward zero mean predictions.

Before applying the SE model to longitudinal panel data it needs to be adapted slightly. Instead of regularizing toward zero, we regularize toward the person-specific mean. This is easily achieved using the established combination rules for GPPMs. One simply adds the GPPM representation of the random intercept model to the SE model. This results in the following random intercept SE model

$$\begin{array}{l} Y_{i}\left(t\right)\sim GP\left(\mu_{I},\sigma_{I}^{2}+k_{se}\left(t,t^{\prime }\right)+\delta \left(t-t^{\prime }\right)\sigma_{\epsilon }^{2}\right). \end{array}$$

The random intercept SE model, which we will abbreviate to SE model in the remainder, achieves, as expected, a substantively better interpolation performance (NLPP = 2.33). This almost perfect interpolation performance is also apparent in the visualization of the predictive distribution for one person in Figure 2B.

The SE model also extrapolates better than the LGCM (NLPP = 11.89). This seems to be caused by the SE model increasing the variance of the predictive distribution for data points far away from the training data whereas the variance of predictive distribution from the LGCM remains almost constant (compare Figures 2A,B). As a consequence, the LGCM makes wrong predictions with high confidence for the data points far away from the training data. However, in contrast to the extrapolation performance of the SE model, the interpolation performance can still be considerably improved, as is visible in Figure 2B. Essentially, the SE model falls back to a constant predictive distribution centered around the person-specific mean with a large variance.

This observation motivates the development of a class of hybrid models that consist of a combination of a parametric model, such as the LGCM, and a flexible nonparametric statistical learning models, such as the SE. Such models can also be motivated using more theoretical arguments. Within-person trajectories are often conceptualized as consisting of a combination of intraindividual change and intraindividual variability (Nesselroade, 1991; Ram and Grimm, 2015). Intra-individual change is believed to reflect the true change and is characterized by a relatively slow, well-behaved trajectories; whereas intraindividual variability is believed to occur at a much smaller time scale and is believed to reflect more chaotic, short-lived fluctuations around the intraindividual change. The hybrid of a parametric model and a flexible statistical learning model seems perfectly suited for this situation. The parametric part captures the long-term intraindividual changes, whereas the flexible nonparametric part captures the intraindividual variability. The random intercept SE is also a hybrid model as it combines the parametric random intercept with the nonparametric SE model.

We demonstrate the utility of such models using the LGCM+SE model as an example. Importantly, GPPM would also allow the parametric model to be a more complex model such as the “exponential rise to the limit model.” Mixing those two models, leads to the following model

$$\begin{array}{l} Y_{i}(t)\sim GP(\mu_{I}+\mu_{S}t,\;\sigma_{I}^{2}+\;t\sigma_{S}^{2}t^{\prime }+\sigma_{IS}(t+t^{\prime })\\ \;+\delta (t-t^{\prime })\sigma_{\in }^{2}k_{se}(t,t^{\prime })+\;\delta (t-t^{\prime })\sigma_{\in }^{2}). \end{array}$$

Effectively, this model regularizes the SE model using the LGCM. Thus, it falls back to a LGCM in regions with few data and to a SE model in regions with much data. As a result, it essentially behaves like the flexible SE in regions with many training samples and is thus able to fit a large class of functions, in these regions. In regions with no training samples, it behaves like the LGCM (with larger predictive variance to reflect for the presence of intraindividual variability), and thus might be better at extrapolation.

The example data are generated as a combination of a LGCM and an unknown deviation from the LGCM.

$$\begin{array}{l} Y_{i}\left(t\right)=\underset{\text{LGCM}}{\underset{︸}{I_{i}+S_{i}t+\epsilon_{i}\left(t\right)}}+\underset{\text{deviation}}{\underset{︸}{f\left(t;\theta_{i2}\right)}}\\ \;\left[\begin{array}{c} I_{i}\\ S_{i} \end{array}\right]\sim N\left(\left[\begin{array}{c} \mu_{I}\\ \mu_{S} \end{array}\right],\left[\begin{array}{c} \sigma_{I}^{2}\sigma_{IS}\\ \sigma_{IS}\sigma_{S}^{2} \end{array}\right]\right),\\ \;\sigma_{i2}\sim N\left(\mu_{\theta_{2}},\Sigma_{\theta_{2}}\right),\epsilon_{i}\left(t\right)\sim GP\left(0,\delta \left(t-t^{\prime }\right)\sigma_{\epsilon }^{2}\right) \end{array}$$

This can be interpreted as the intraindividual long-term change being appropriately represented by a LGCM but no or little knowledge is present about the short-term intraindividual variability. As parameter value for the LGCM, we used $\mu_{I}=0,\mu_{S}=3,\sigma_{I}^{2}=20,\sigma_{S}=5,\sigma_{IS}=2,\sigma_{\epsilon }^{2}=1$. For the deviation term, we used $f\left(t;\varphi_{i}\right)=\frac{1}{2}t\cos\left(2\pi \frac{1}{10}t-\varphi_{i}\right)$ with $\varphi_{i}\sim N\left(0,4\pi^{2}\right)$, which corresponds to an oscillation with person-varying phase and time-varying (increasing) amplitude. For creating the training, the test, and the interpolation sets, we used the same time points and numbers of persons as before.

**Figure 3:**
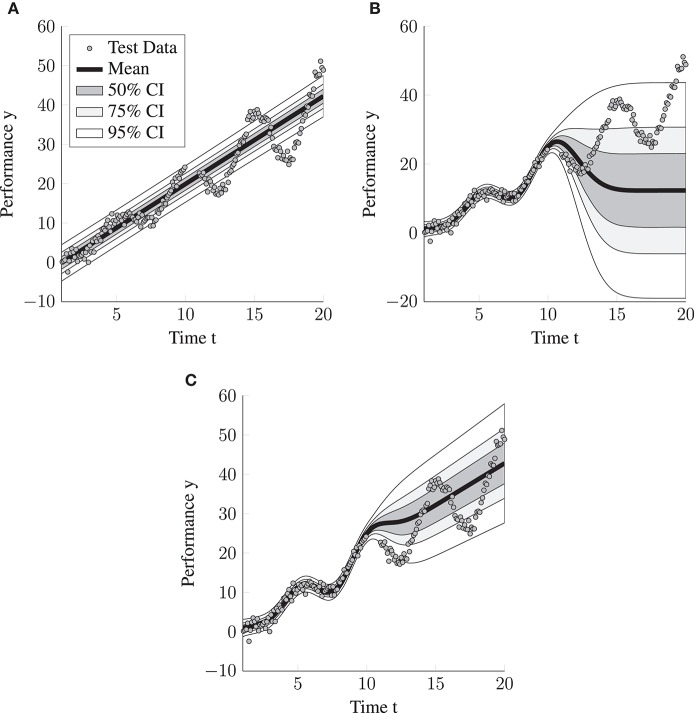
Visualization of the predictive distributions on the data generated from the LGCM + unknown deviation distribution for the three considered models. CI = credibility interval. **(A)** LGCM. **(B)** SE Model. **(C)** LGCM+SE.

We compared the performance of the LGCM, the SE and the LGCM+SE model (see Table 2 for the full results). Overall, the LGCM+SE model performs best. With regard to the interpolation performance (NLPP = 2.87) it performs relatively close to the expected optimal performance, whereas the extrapolation performance is far from optimal (NLPP = 30.275). The SE is, as expected, less accurate than the LGCM+SE model in extrapolation (NLPP difference is 1.36) and has only a slight advantage over the LGCM+SE model in terms of the interpolation performance (NLPP difference is 0.02). The difference for the improved interpolation performance is caused by the LGCM+SE model regularizing toward the LGCM, so a person-specific linear trajectory instead of a person-specific mean (compare Figures 3B, C). The LGCM, as expected, performs much worse than the former two. The interpolation performance is reduced by the lacking flexibility of the LGCM (NLPP = 74.968), whereas the extrapolation performance (NLPP = 299.14) is diminished by the LGCM adapting its uncertainty, as expressed by the predictive variance, too slow (see Figure 3A).

**Table 2:** Negative log predictive probabilities on the data generated from the LGCM + unknown deviation distribution for the compared models as estimated by the different test sets.

**Model**	**Interpolation**	**Extrapolation**	**Combined**
LGCM	74.97	299.14	375.37
SE Model	2.85	31.641	34.278
LGCM+SE Model	2.87	30.275	33.038

While we expect hybrid models such as LGCM+SE to perform best in situations where intraindividual change and intraindividual variability are present, and the parametric model for the intraindividual change is correctly specified, we also expect the hybrid models to perform almost as well as the flexible statistical learning models even if the parametric model is completely misspecified. The reason for this is that they essentially inherit the ability of the flexible statistical learning model to fit most functional forms and thus to achieve near-optimal interpolation performance. We demonstrate this by applying the LGCM+SE model to the data from the “exponential rise to the limit model.” The LGCM+SE model achieves near-optimal interpolation performance (see, also Figure 2C). The interpolation performance of the LGCM+SE model was even slightly better than that of the SE model (NLPP difference 0.12). Note that this near-optimal performance is only achieved in the time span where many training data points are available (interpolation). The extrapolation performance, in contrast, is far from optimal. This reveals that the predictive accuracy of the flexible machine learning models does not primarily depend on the number of measurements per person or the number of persons. Instead, the person-specific predictions obtained for person *i* and time point t^*^, are most accurate when an observation *y*_*it*_ is available where the time point *t* is close to the time point t^*^. Thus, the flexible machine learning models work best for interpolation. However, as we showed, they still may extrapolate better than classical psychometric models.

### 6.2. Real Data: Smooth Models

We demonstrate that the hybrid random intercept SE model, which is one of the models uniquely representable by GPPM, is a suitable alternative to models routinely used in psychological research. This is the case considering the statistical learning as well as the explanatory perspective. We demonstrate this by showing that the random intercept model leads to more accurate predictions (statistical learning perspective) as well as a higher model probability (explanatory perspective) of the model compared to the continuous-time random intercept autoregressive model of order 1, which was previously used to analyze the example data set.

We start with the observation that the random intercept SE is very similar to a popular model used in psychological research, the continuous-time random intercept autoregressive model of order 1. The *n* = 1 continuous-time autoregressive model of order 1 is the Ornstein-Uhlenbeck process, which is one particular Gaussian process. The stationary Ornstein-Uhlenbeck process has mean function and kernel as follows

$$\begin{array}{l} m\left(t;\theta \right)=\mu_{I},\;k\left(t,t^{\prime };\theta \right)=\sigma_{se}^{2}\exp\left(-\frac{\left|t-t^{\prime }\right|}{l}\right). \end{array}$$

To use this model for *n* > 1 data, it is typically extended with a random intercept, which leads to the continuous-time version of the random intercept autoregressive model of order 1, which has the following GPPM representation

$$\begin{array}{l} m\left(t;\theta \right)=\mu_{I},\;k\left(t,t^{\prime };\theta \right)=\sigma_{I}^{2}+\sigma_{se}^{2}\exp\left(-\frac{\left|t-t^{\prime }\right|}{l}\right). \end{array}$$

Comparing the kernel functions of the random intercept SE model and the random intercept continuous-time autoregressive model reveals that in the former the within-person autocorrelation is assumed to decline according to a squared exponential and for the latter according to an exponential.

**Figure 4:**
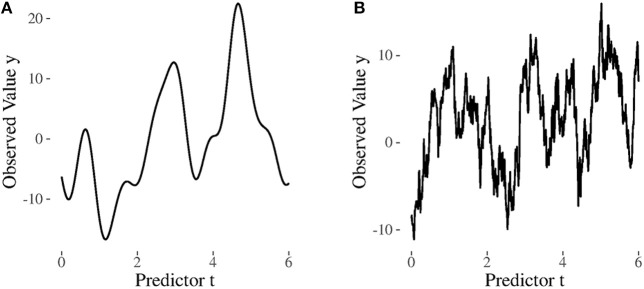
Graphical illustration of the differences between the squared exponential and the exponential kernel. Example trajectories implied by each kernel are shown. To generate the data, the variance parameter was set to $\sigma_{se}^{2}=2$ and the length scale parameter to *l* = 1 for the exponential and to *l* = 0.25 for the squared exponential kernel. **(A)** Squared exponential. **(B)** Exponential.

Despite their mathematical similarity, there is a substantial difference between the exponential and the squared exponential kernel. Both kernels are special cases of the so-called Matérn kernel (Schulz et al., 2018). From a Matérn kernel perspective, they represent two endpoints on a continuum (Schulz et al., 2018): The squared exponential kernel implies very smooth (that is infinitely differentiable) trajectories, whereas the exponential kernel implies rather unsmooth, rough, trajectories. In Figure 4, we visualize this difference by contrasting a trajectory generated from a squared exponential kernel with a trajectory generated from an exponential kernel.

On a more conceptual level, smoothness can be regarded as the mathematical implementation of the “nature does not jump” assumption, which implies that changes in nature typically do not occur abruptly, and this has already been proposed as a fundamental principle in nature by, for example, Darwin (1859) and Leibniz (1704). The rough trajectories implied by the exponential model, on the other hand, are not in line with this assumption. Thus, if the “nature does not jump” assumption is fulfilled using a model that implements it should lead to better predictions (statistical learning perspective) and better parameter estimates (explanatory perspective).

To investigate the usefulness of the random intercept squared exponential (SE) model (from now on simply called SE model) for psychological data analysis, we reanalyzed data that have previously been analyzed using a continuous-time autoregressive model (Voelkle et al., 2012). The data originate from a German panel study (Heitmeyer, 2004), measuring people aged 16 years and older who do not have an immigration background using computer-assisted interviews. Measurements were performed in 2002, 2003, 2004, 2006, and 2008, but not in 2005 and 2007.

Among other variables, authoritarianism was measured, which reflects a person's preference to submit under authorities, to orient along with the perceived conventions of the in-group, and to aggressive stances toward outgroups. For illustrative purposes, we will focus on this measure in the following. A total of *n* = 2, 722 people took part in the first wave of the study, with considerable drop out over time (see Voelkle et al., 2012, for details).

To investigate whether the SE model should be preferred over the exponential model, we used an explanatory as well as a statistical learning model selection procedure. For the explanatory procedure, we compared the models based on the Bayesian information criterion. We did not use the likelihood-ratio test because the two models are not nested. As the statistical learning procedure, we assessed the predictive performance of the models using cross-validation. As the splitting strategy, we split by persons. More specifically, we used leave-one-person out cross-validation. This estimates the ability of the models to predict trajectories of previously unseen persons. As before, we used the negative log predictive probability as loss functions.

As can be seen in Table 3, the prediction inaccuracy as measured by the negative log predictive probability as well as the BIC were both lower for the SE model. The Bayesian information criterion values can be translated into model posterior probabilities (Wagenmakers and Farrell, 2004). The obtained values translate into a probability of >0.99 that the SE model is the better model for this dataset. Note, however, that this is merely a measure of relative model fit and cannot be interpreted as measure of absolute fit.

**Table 3:** Bayesian information criterion (BIC), and negative log predictive probability (NLPP) for the exponential and the SE model.

**Measure**	**Exponential model**	**Squared exponential model**
BIC	10876.14	**10850.39**
NLPP	10846.54	**10821.99**

*Bold face marks the model selected on the basis of the corresponding measure. The smaller value of a measure indicates which model to select*.

After having established that the SE model should be preferred, we investigate the impact of using the traditional exponential model instead on both explanatory and statistical learning results.

We start with the explanatory perspective. The parameter estimates and their corresponding 95%-confidence intervals are displayed in Table 4. We focus an all parameter but the length scale parameter *l* and the variance parameter $\sigma_{se}^{2}$ as they implement different concepts across the two models. The estimated mean function, as represented by the intercept parameter μ_*I*_, is identical across both models. In contrast, all remaining parameters are different. For example, in the exponential model, the confidence interval for the intercept variance contains 0, whereas it does not for the SE model. Thus, using a classical hypothesis testing approach, one could only conclude that there are no significant differences with regard to the starting level of authoritarianism across persons, that is, the null hypothesis of no differences in starting level cannot be rejected. However, the preferred SE model indicates significant differences in the starting levels across persons.

**Table 4:** 95% confidence intervals as well as maximum likelihood estimates for the parameters from exponential and the squared exponential model.

**Parameter**	**Lower bound**	**Estimate**	**Upper bound**
**Exponential model (auto-regressive model)**			
μ_*I*_	2.82	2.85	2.87
$\sigma_{I}^{2}$	0.00	0.00	0.11
$\sigma_{se}^{2}$	0.37	0.47	0.50
*l*	13.24	13.42	15.26
$\sigma_{\epsilon }^{2}$	0.04	0.05	0.06
**Squared exponential model**			
μ_*I*_	2.82	2.85	2.87
$\sigma_{I}^{2}$	0.21	0.26	0.30
$\sigma_{se}^{2}$	0.16	0.19	0.23
*l*	20.95	21.39	30.54
$\sigma_{\epsilon }^{2}$	0.07	0.08	0.08

**Figure 5:**
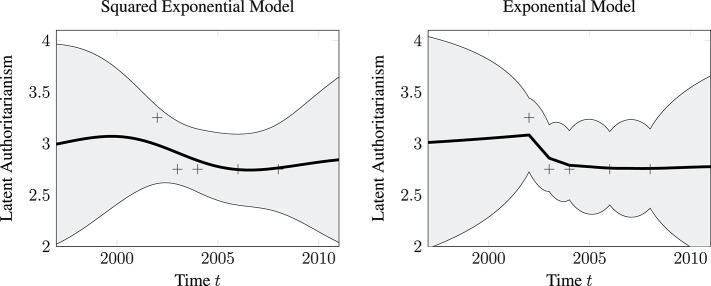
Person-specific predictions of the squared exponential and the exponential model for one randomly selected person. The bold line indicates the mean of the predictive distribution for every time point. The gray area displays the 95% credible region. Crosses depict observed training data.

For the statistical learning perspective, we investigate the impact of the model choice on the predictive distribution. We have already seen that the predictive distribution of the SE model is more accurate, as quantified by the lower cross-validated negative log predictive probability. We now also compare the two predictive distributions visually. In Figure 5, we show the predictive distribution obtained for one exemplary person. We plot the predictive distribution only for latent authoritarianism, that is, the authoritarianism score without being contaminated by measurement error. The most notable difference between the two predictive distributions is that the predictive mean, as well as the predictive variance, is smooth for the SE model, whereas it is not for the exponential model. This again implements the “nature does not jump” assumption.

## 7. Summary and Discussion

In the present paper, we have introduced Gaussian process panel modeling (GPPM), an extension of Gaussian process regression (GPR) for the analysis of panel data. GPPM provides great flexibility in specifying parametric models, nonparametric models, or combinations of both. It offers a choice of two inference frameworks focusing on either explanation or prediction. It subsumes many standard modeling approaches for longitudinal data such as linear structural equation models and state-space models as special cases but also extends the space of expressible models beyond those common approaches. To make GPPM available, we provide the R package “gppm” (Karch, 2018).

GPPMs are specified by a kernel language consisting of a mean function and a kernel. Throughout this manuscript, we have demonstrated how the flexibility of the kernel-language and its combination rules can be used to specify novel panel data models (in Appendix 1, we provide an overview of the models used). Specifically, we used GPPM to express hybrid models such as the random intercept squared exponential (SE) model and the LGCM+ SE model. In a simulation study, we showed that the LGCM+SE model combines the advantages of the parametric LGCM and the nonparametric statistical learning SE model. The random intercept SE model was also featured in the empirical illustration, in which it was shown to be a viable alternative to the popular random intercept autoregressive model (Hamaker et al., 2015) when smoothness of the process is a reasonable prior belief.

Regarding inference procedures, the frequentist inference procedures for GPPM enable explanatory data analysis, which aims to recover the population distribution. The Bayesian statistical learning inference procedures provide a predictive modeling perspective that is relatively uncommon in the analysis of panel data. As demonstrated in this article, one advantage of the statistical learning inference framework is that its conclusions about the predictive accuracy of a model are also valid when the model is not correct. It is often unrealistic to assume that a chosen model is correctly specified, and adopting a predictive modeling perspective that does not rely on this assumption may be beneficial. Additionally, GPPM allows operating with hybrid models that are partly informed by theory and partly informed by data with a specific focus on maintaining generalization performance and avoiding overfitting (see also, Brandmaier et al., 2016). Thus, we believe that the statistical learning inference perspective provides a viable addition to the methodological toolbox for analyzing panel data. Because all Bayesian quantities can be obtained analytically, statistical learning inference in GPPM is exact and faster than in commonly used Markov chain Monte Carlo-based approaches.

With the breadth of models that GPPM can represent, where does GPPM land on the confirmatory-exploratory spectrum? With confirmatory analysis, we refer to any analysis where hypotheses are deducted from theory and are tested with all parameters of the GPPM defined before data were seen. An exploratory analysis is performed without any clear theory-driven hypothesis in mind and, thus, no a priori fixed kernel or mean function. Clearly, GPPM can be used for both approaches. Confirmatory testing requires one to specify one kernel and mean function and, then, infer their parameters from empirical data. Exploratory approaches may entail those where a flexible kernel (such as the SE kernel) is employed to maximize predictive accuracy (but it may have a low potential for explanation) or where kernels are successively expanded to improve predictive accuracy (for example, by augmenting a LGCM kernel with inadequate fit with a SE kernel). For exploratory approaches, model selection between all candidate models needs to be done with caution, such that, ideally, the same data should not be used for either model fitting, model selection, or performance evaluation^2^, or as an alternative approach, appropriate cross-validation strategies (such as nested CV, see, Karch et al., 2015) can be employed.

Within the psychometric modeling community, there have been many previous efforts to provide robust inference on covariance models both within the frequentist (e.g., Satorra, 1990; Bollen et al., 2007) and the Bayesian inference framework (e.g., Lee and Xia, 2008). In contrast to our work, these approaches retain the focus on explanatory modeling. Most approaches (e.g., Satorra, 1990; Lee and Xia, 2008) focus on robustness concerning outliers or distributional assumptions. Other approaches go beyond this and consider more serious misspecification, which is known as structural misspecification (Satorra, 1990). However, the structural misspecification considered is often relatively mild. For example, Bollen et al. (2007) investigate structural misspecification in the sense of a few paths missing from a factor model, while the majority of the model is correctly specified. In contrast to this, the statistical learning inference approach, which estimates how well a certain model predicts, is valid under all forms of misspecification (Breiman, 2001).

### 7.1. Extensions, Limitations and Future Research Directions

For lack of space, we only briefly hint at some further opportunities for modeling with GPPM that may be useful in practice. Correlated error structures can be implemented by using the appropriate kernel instead of the white noise kernel $\delta \left(t-t^{\prime }\right)\sigma_{\epsilon }^{2}$. The autoregressive error structure, for example, is represented by the autoregressive kernel displayed in Equation (14). Time-varying errors can be implemented using the same approach. For example, a linear increase in measurement error is implemented by $\delta \left(t-t^{\prime }\right)\left(\sigma_{1}^{2}+\sigma_{2}^{2}t\right)$. Representing more complex hierarchies beyond the simple two-level model with observations nested in persons is also possible in GPPM. We demonstrate this in Appendix 2.

One current limitation of GPPM is that random effects can only be specified for linear parameters of the mean function. Consequently, multiplicative random effects (Ram and Grimm, 2015) or random effects on kernel parameters, needed to implement probabilistic person-varying measurement error, can currently not be implemented. Deterministic person-varying measurement error can already be implemented. GPPM can be extended to allow for random effects for all parameters. However, we expect that with this extension exact inference is not possible anymore, and one has to fall back to approximate inference, similar to other approaches allowing for random effects on all parameters (c.f. Asparouhov et al., 2017; Driver and Voelkle, 2018).

Specifying a multivariate GPPM is possible given our current framework, but it may appear more intricate than in standard state-space modeling and structural equation modeling specification. Beyond the kernel for the auto-covariance of each variable, we also need cross-covariance kernels for each pair of variables (Alvarez et al., 2010).

GPPM, as introduced in the present paper, is limited to continuous data. To extend GPPM to nominal or ordinal data, one can build on a rich library of methods developed for extending GPR. Just like in generalized linear models, so-called link functions (Rasmussen and Williams, 2006, Chapters 3 and 9.3) are used to model non-Gaussian observations. Using the same approach, non-normal measurement error for continuous data, for example, Laplace errors as commonly used in robust methods, can also be implemented. As in other extensions of linear models to accommodate non-Gaussian observations, these generalizations complicate inference. However, the appropriate algorithms have already been developed (Rasmussen and Williams, 2006) and await to be adapted to GPPM.

GPPM generalizes all methods that are restricted to Gaussian processes and use either frequentist or statistical learning inference. While this subsumes many methods, this excludes methods that imply non-Gaussian stochastic processes at the latent level, such as nonlinear structural equation modeling (Jöreskog and Yang, 1996) or nonlinear state-space modeling (Chow and Zhang, 2013).

With regard to frequentist model selection, many different approaches beyond AIC, and BIC can be adapted for GPPM (Burnham, 2013). One promising approach is the minimum description length principle (Grünwald, 2007); in particular, normalized maximum likelihood (Myung et al., 2006).

A main contribution of this work is explicitly drawing the connection of the field of kernel methods to the analysis of longitudinal data in psychological research. This opens multiple opportunities for future research: Besides the squared exponential model we have emphasized here, many other GPR models can be readily applied to panel data (Roberts et al., 2013; Duvenaud, 2014). Among the most promising candidates are periodic models (Rasmussen and Williams, 2006, Chapter 4), and change-point detection models (Turner, 2012), which could be viable alternatives to their existing state-space equivalents (Chow et al., 2005, 2018).

When appropriately safeguarding against overfitting, exploratory analysis has many opportunities for the analysis of panel data and can profit from research in kernel methods. One generic approach is to define a model that is flexible enough to fit most functions given enough training data. The squared exponential kernel we introduced is a prototypical example. However, exploratory analysis has been taken one step further by an algorithm that automatically learns the kernel from data and then describes the model in natural language (Lloyd et al., 2014). This algorithm also exploits the fact that complex models can be specified by combining a small set of base models, as we also discussed in this paper. Extending this algorithm for use in GPPM would result in a method that learns the between- and the within-person model from empirical data. This approach has the potential to find better models than the current practice of searching for a model by heuristics or merely relying on default models. Future research will have to address the right trade-offs between bias (over/underfitting) and variance (model selection uncertainty) in applying such automated model searches and how and to what extent prior knowledge can be incorporated in this model search.

Speeding up model-fitting algorithms for panel data models is becoming increasingly important as technological progress enables obtaining unprecedented amounts of data at little cost. Especially, fitting structural equation models on intensive longitudinal data will become a problem as the time required for parameter estimation grows cubically with the number of time points due to the necessary inversion of the model-implied covariance matrix. The same is true for GPPM. However, for GPPM one can adapt faster approximative inference algorithms, which have been developed for GPR (for example, Lawrence et al., 2003; Leithead and Zhang, 2007; Hartikainen and Särkkä, 2010). These promise to speed up inference in GPPM, and consequently, longitudinal structural equation models substantially. Future work needs to investigate the speed-accuracy trade-off for inference when resorting to these approximations.

GPPM promises to deepen our understanding of the close connections between different families of models and modeling approaches. Specifically, while we have demonstrated that GPPM generalizes linear structural equation modeling, and linear state-space modeling, it has also been shown that GPR subsumes smoothing splines, (kernel) ridge regression, Bayesian (kernel) regression, and it is closely related to other Machine Learning methods such as support vector machines and (deep) neural networks (Rasmussen and Williams, 2006; Lee et al., 2017). One interesting result that follows from the identification of structural equation modeling as a special case of GPPM, and GPPM's close relation to Bayesian kernel regression, is that every conventional structural equation model is equivalent to Bayesian linear regression in some high-dimensional space. We believe that making these connections explicit has the potential to foster innovations from seemingly distant research areas, such as kernel learning or deep learning, for the analysis of psychological data. In this regard, we share the hope of Yarkoni and Westfall (2017) that the predictive modeling approach is regarded as an opportunity, not a threat, and psychological researchers equipped with a mix of classical and new methods will have a higher likelihood of finding the appropriate modeling and inference framework for their research question.

## Data Availability Statement

Publicly available datasets were analyzed in this study. This data can be found here: http://dx.doi.org/10.1037/a0027543.supp.

## Author Contributions

JK developed the method, performed the computations, and wrote the first draft of the manuscript. AB and MV supervised the project. All authors discussed the results and contributed to the final manuscript.

### Conflict of Interest

The authors declare that the research was conducted in the absence of any commercial or financial relationships that could be construed as a potential conflict of interest.

## Footnotes

^1^Technically, a realization of a stochastic process is of infinite size as the stochastic process contains infinitely many random variables. So, technically *y*_*i*_ is a realization of a finite subset of the random variables contained in the stochastic process.

^2^These subsets of data are then often referred to as training set, validation set, and test set.
