# Supplementary material for: Gaussian Process Panel Modeling—Machine Learning Inspired Analysis of Longitudinal Panel Data
Source: Front Psychol. 2020 Mar 19;11:351. doi: 10.3389/fpsyg.2020.00351 (PMC7096578; doi:10.3389/fpsyg.2020.00351)
Supplement: Supplementary file 1 [file Data_Sheet_1.pdf]

Appendix for Gaussian Process Panel Modeling — Machine Learning Inspired Analysis of Longitudinal Panel Data

1 EXEMPLARY GAUSSIAN PROCESS PANEL MODELS

In Table S1, we present a selection of models expressible in Gaussian process panel modeling (GPPM) with corresponding mean function and kernel. For brevity, all models are shown without measurement error, which crucially needs to be added when applying these models. For white noise Gaussian error, add $\delta(t-t')\sigma_\epsilon^2$ to the kernel. For simplicity, we assumed no covariance between the intercept and the slope terms for the quadratic latent growth curve model (LGCM). The quadratic LGCM can be further extended to polynomials of arbitrary degree p. The mean function and kernel representing a polynomial of degree p are $m(t;\theta) = \sum_{i=0}^{p} \mu_i t^i$ and $k(t,t';\theta) = \sum_{i=0}^{p} t^i \sigma_i^2 t'^i$.

Name	Mean Function	Kernel
	Psychometric Models	
Random intercept	μ_I	σ_I^2
Random intercept, fixed Slope	$\mu_I + \mu_S t$	σ_I^2
Linear LGCM	$\mu_I + \mu_S t$	$\sigma_I^2 + t\sigma_S^2 t' + \sigma_{IS}(t+t')$
Quadratic LGCM	$\mu_I + \mu_S + \mu_{S_2} t^2$	$\sigma_I^2 + t\sigma_S^2 t' + t^2 \sigma_{S_2}^2 t'^2$
Random Intercept AR(1)	μ_I	$\sigma_I^2 + \sigma_e^2 \exp\left(-\frac{\lvert t-t'\rvert}{l}\right)$
	Statistical Learning Models	
Ridge Regression	0	$x^\top \sigma_b^2 x'$
SE Model	0	$\sigma_{se}^2 \exp\left(-\frac{\lvert\lvert x-x'\rvert\rvert^2}{l}\right)$
	Hybrid Models	
Random Intercept + SE	μ_I	$\sigma_I^2 + \sigma_{se}^2 \exp\left(-\frac{(t-t')^2}{l}\right)$
LGCM + SE	$\mu_I + \mu_S t$	$\sigma_I^2 + t\sigma_S^2 t' + \sigma_{IS}(t+t') + \sigma_{se}^2 \exp\left(-\frac{(t-t')^2}{l}\right)$
Exponential Decay + SE	$\mu_b + \mu_d \exp(-ts)$	$\sigma_b^2 + \sigma_d^2 \exp\left(-(t+t')s\right) + \sigma_{se}^2 \exp\left(-\frac{(t-t')^2}{l}\right)$

Table S1. Mean functions and kernels for exemplary Gaussian process panel models. LGCM = linear growth curve model, AR(1) = autoregressive model of order 1, SE = squared exponential,

For the statistical learning models, the number of predictors is typically quite high, which is why we use x, which denotes a vector of predictors, instead of t, which denotes one single predictor, namely time. There are many more statistical learning methods that can be expressed as a Gaussian process regression (GPR) and thus as a GPPM. Presenting their mean and kernel function goes beyond the scope of this text. We refer the interested reader to Seeger (2002) and Rasmussen and Williams (2006, Chapter 6.3) for smoothing splines; Duvenaud et al. (2011) for generalized additive models; and Lee et al. (2017) for (deep) neural networks.

Mixing the statistical learning models and the longitudinal psychological models lead to the two hybrid models that we discuss in detail in the illustration section. Here, we also present the exponential decay + squared exponential (SE) model, a hybrid model whose parametric part implies the nonlinear "exponential rise to the limit" trajectories.

2 MORE COMPLEX HIERARCHIES

To showcase the ability of GPPM to represent more complex hierarchies, we demonstrate how to specify the longitudinal version of the two-way cross-classified model introduced in Leckie (2013). The model expressed using the multilevel notation is as follows:

$$Y_{ijk}(t) = \beta_0 + v_k + u_j + \epsilon_{ijk}(t), \text{ with } v_k \sim \mathcal{N}(0, \sigma_v^2), u_j \sim \mathcal{N}(0, \sigma_u^2), \epsilon_{ijk}(t) \sim \mathcal{GP}(0, \sigma_\epsilon^2)$$

where $Y_{ijk}(t)$ is the age t score of student i who attended primary school j and secondary school k, β_0 is the mean score across all schools, v_k is the effect of secondary school k, u_j is the effect of primary school j, and $e_{ijk}(t)$ is the student-time level residual error term. The random effects and residual errors are assumed independent of one another. Scores are thus nested within students. Students are nested within primary and secondary schools but primary schools are not nested in secondary schools or vice versa.

If we assume that we observe predictors time t, primary school index j, and secondary school index k, the resulting GPPM is

$$Y_{ijk}(t) \sim \mathcal{GP}(\beta_0, \delta(k-k')\sigma_v^2 + \delta(j-j')\sigma_u^2 + \delta(t-t')\sigma_\epsilon^2).$$

REFERENCES

Duvenaud, D. K., Nickisch, H., and Rasmussen, C. E. (2011). Additive Gaussian Processes. In *Neural Information Processing Systems 2011*, eds. J. Shawe-Taylor, R. S. Zemel, J. C. Bartlett, F. Pereira, and K. Weinberger (Red Hook, NY: Curran Associates)

Leckie, G. (2013). Module 12: Cross-Classified Multilevel Models. In *LEMMA VLE, University of Bristol, Centre for Multilevel Modelling.*

Lee, J., Bahri, Y., Novak, R., Schoenholz, S. S., Pennington, J., and Sohl-Dickstein, J. (2017). Deep Neural Networks as Gaussian Processes. *arXiv preprint arXiv:1711.00165*

Rasmussen, C. E. and Williams, C. K. I. (2006). *Gaussian Processes for Machine Learning* (Cambridge, MA: MIT Press)

Seeger, M. (2002). *Relationships between Gaussian Processes, Support Vector Machines and Smoothing Splines*. Technical report, Institute for Adaptive and Neural Computation (ANC), University of Edinburgh, UK
